# The expression and function of toll-like receptors (TLRs) in ovarian cancer cell lines

**DOI:** 10.1186/2051-1426-3-S2-P76

**Published:** 2015-11-04

**Authors:** Shin-Wha Lee, In-Kyung Lee, Sumin Kim, Sang-Eun Lee, Ha-Young Lee, Dong-Woo Kang, Yong-Man Kim

**Affiliations:** 1 ASAN Medical Center, Seoul, Republic of Korea; 2 ASAN Institute for Life Sciences, Seoul, Republic of Korea

## Objective

This study is to investigate which Toll Like Receptors (TLRs) are expressed on human ovarian cancer cell lines and to evaluate the effect of TLR expression on the growth of ovarian cancer cells.

## Methods

Four human ovarian epithelial carcinoma cell lines SKOV3 (clear), OVCAR3 (serous papillary), SNU8 (serous papillary) and SNU251 (endometrioid) were used for the experiments. To find whether the expression of TLRs is functional on human ovarian cancer cell lines, cytokines (IL-6, IL-10, TNF-α, IL-12, and IFN-γ) were investigated. For statistical analysis, one-way ANOVA with Tukey's multiple comparison post-test was carried out.

## Results

In SKOV3 cell line, TLR 1-7 were expressed and among them, TLR-2, -3 and -5 have strong intensity; however, TLR 9 was absent and TLR 8 was weak. In OVCAR3 cell line, TLR 3 and TLR 5 were much more intense, and TLR-1, -2, -4, -7 and -8 were weak, while TLR 6 and TLR 9 were absent. Unlike other cell lines, SNU8 has strong intensity through TLR 1-9 although TLR9 was a little weaker than the others. In SNU251 cell line, TLR 1-5 and TLR 7-8 were expressed and TLR 6 and TLR 9 were absent or weak. The exposure of cells to ligands for TLR 1-9 showed significant upregulation of IL-6, especially in TLR 5. IL-10 after stimulating TLR 5 was significantly increased in SKOV3 and OVCAR3. Another proinflammatory cytokine TNF-α was assessed, and the result that only TLR 5 in OVCAR3 has a significant induction. IL-12p70 on TLR 9 was induced in the four cell lines but the result didn't show any significance and IFN-γ was induced insignificantly in SKOV3 and OVCAR3 for TLR 9 only.

## Conclusion

The expression and biologic function of TLRs are different in each ovarian cancer cell line. In order to target TLRs for the management of ovarian cancer, further studies are needed on the effects of TLRs in tumor development.

